# Supplementary material for: Significant distinct branches of hierarchical trees: a framework for statistical analysis and applications to biological data
Source: BMC Genomics. 2014 Nov 19;15(1):1000. doi: 10.1186/1471-2164-15-1000 (PMC4253613; doi:10.1186/1471-2164-15-1000)
Supplement: Supplementary file 1 — Additional file 1: A time complexity and performance analysis, Figure S1, Figure S2 and Table S1. A PDF file containing 1) a comparison of time complexity and performance for TBEST, SC, SLB and DTC, 2) Figure S1, an 11-panel figure illustrating null distribution of tightness, 3) Figure S2, a comparison of empirical p-value estimates for tightness to EVT-based estimates and 4) Table S1, detailing the properties of the Simulated6 dataset. (PDF 462 KB) [file 12864_2014_6704_MOESM1_ESM.pdf]

Additional File 1

Comparative analysis of time complexity and performance 1
1. Time Complexity 1
2. Performance 2
References 3
Figure S1 4
A: Simulated6 4
B: Simulated6 4
C: Leukemia 5
D: Leukemia 5
E: T10 6
F: T10 6
G: Organelles 7
H: Organelles 7
I: Chondrosarcoma 8
J: Chondrosarcoma 8
K: Chondrosarcoma 9
Figure S2 10
Table S1 11

Comparative analysis of time complexity and performance

1. Time Complexity

Time complexities of TBEST and other three existing methods are listed in Table S1.1. These depend on the number of randomizations m, number of observations n and number of variables d. For the two top-down methods, SC and SLB, the complexities as stated correspond to the worst-case scenario wherein all internal nodes of the tree are examined.

Table S1.1 Time complexities of TBEST and of the published methods

TBEST	$O(mn^2d) + O(mn^3)$
SC	$O(mn^2d) + O(n^3d)$
SLB	$O(mn^3d) + O(mn^4)$
DTC	$O(n^2d)$

TBEST requires, for each randomization, to compute a dissimilarity matrix at a cost $O(n^2d)$ and to grow a hierarchical tree at a cost $O(n^3)$. Other computational costs, such as computing the tightness, are sub-dominant to these two.

The complexity of SC was computed under the worst-case assumption that one of the daughters at each internal node of the tree is a single leaf. With this assumption, computing statistic on n-1 hierarchies with each simulation, from 1 to m, needs $O(mn^2d)$. For each branch, from 1 to n-1, SC computes variance-covariance matrix [2], which takes $O(n^3d)$ in total. Other computational costs, such as computing the eigenvalues of the variance-covariance matrix, are sub-dominant to these two.

SLB performs randomization for each internal node being examined and requires computation of dissimilarity matrix and hierarchical clustering for each such randomization [3]. The necessity of performing these operations separately for each internal node explains the additional factor of n in the complexity of SLB compared to that of TBEST.

DTC does not perform statistical assessment of partitions, and its complexity is independent of m. The complexity as stated refers to the worst-case scenario, wherein the minimal allowed number of leaves on a branch is one. The dominant term in the complexity estimate comes from executing step 2 of the dynamic hybrid algorithm [1].

2. Performance

Here we report execution times of TBEST and of the other three methods for all combinations of datasets, dissimilarities and linkages studied in this work. These are reported in five tables, one per dataset.

The following computing resource was used:

MacBook Air	
Processor Name:	Intel Core i5
Processor Speed:	1.3 GHz
Number of Processors:	1
Total Number of Cores:	2
L2 Cache (per Core):	256 KB
L3 Cache:	3 MB
Memory:	4 GB

For TBEST, SC and SLB, 5000 randomizations were performed. For SC and DTC the packages sigclust and dynamicTreeCut were used, respectively [4-5]. For TBEST and SLB the R language package TBEST was invoked [6]. All these packages are publicly available from the Comprehensive R Archive Network (CRAN). The TBEST package facilitates parallel execution, and both cores of the processor were employed.

Table S2.1 Simulated6

Method / Combination	Euclidean dissimilarity complete linkage	(1 - Pearson correlation) dissimilarity average linkage
TBEST	74.00s	123.00s
SC	78.10s	78.10s
SLB	232.83s	122.76s
DTC	0.05s	0.04s

Table S2.2 Leukemia

Method / Combination	Euclidean dissimilarity Ward linkage	(1 - Pearson correlation) dissimilarity average linkage
TBEST	92.36s	118.83s
SC	92.47s	92.47s
SLB	400.42s	129.02s
DTC	0.03s	0.02s

Table S2.3 T10

Method / Combination	Euclidean dissimilarity Ward linkage	(1 - Pearson correlation) dissimilarity average linkage
TBEST	376.92s	446.75s
SC	297.65s	265.15s
SLB	1063.80s	448.84s
DTC	0.07s	0.06s

Table S2.4 Organelles

Method / Combination	(1 - Pearson correlation) dissimilarity Ward linkage	(1 - Pearson correlation) dissimilarity average linkage
TBEST	320.38s	320.22s
SC	2135.00s	2125.91s
SLB	1382.38s	326.29s
DTC	0.01s	0.01s

Table S2.5 Chondrosarcoma

Method / Combination	(1 - Spearman correlation) dissimilarity Ward linkage	(1 - Kendall correlation) dissimilarity average linkage	Manhattan dissimilarity Ward linkage
TBEST	117.84s	121.13s	5.50s
SC	30.01s	17.09s	54.99s
SLB	232.01s	194.40s	24.30s
DTC	0.03s	0.04s	0.02s

References

1. Langfelder P, Zhang B, Horvath S: **Defining clusters from a hierarchical cluster tree: the Dynamic Tree Cut package for R**. *Bioinformatics* 2008, **24**(5):719-720.

2. Liu Y, Hayes DN, Nobel A, Marron JS: **Statistical Significance of Clustering for High-Dimension, Low-Sample Size Data**. *Journal of the American Statistical Association* 2008, **103**(483):1281-1293.

3. Munneke B, Schlauch KA, Simonsen KL, Beavis WD, Doerge RW: **Adding confidence to gene expression clustering**. *Genetics* 2005, **170**(4):2003-2011.

4. Langfelder P, Zhang B, Horvath S: **dynamicTreeCut: Methods for detection of clusters in hierarchical clustering dendrograms**. *The Comprehensive R Archive Network*: http://cran.r-project.org/web/packages/dynamicTreeCut/index.html.

5. Huang H, Liu Y, Marron JS: **sigclust: Statistical Significance of Clustering**. *The Comprehensive R Archive Network:* http://cran.r-project.org/web/packages/sigclust/index.html.

6. Sun G, Krasnitz A: **TBEST: Tree branches evaluated statistically for tightness**. *The Comprehensive R Archive Network*: http://cran.r-project.org/web/packages/TBEST/index.html.

Figure S1

The null distribution of node tightness *S* depends on the number of leaves.

This dependence is illustrated for all the benchmarks and dissimilarity – linkage combinations analyzed. In each case the distributions of *S* are shown for nodes with 2, 5 and 20 leaves. Each plot is based on 5000 randomizations of the respective data set.

A: Simulated6

Euclidean dissimilarity – complete linkage combination

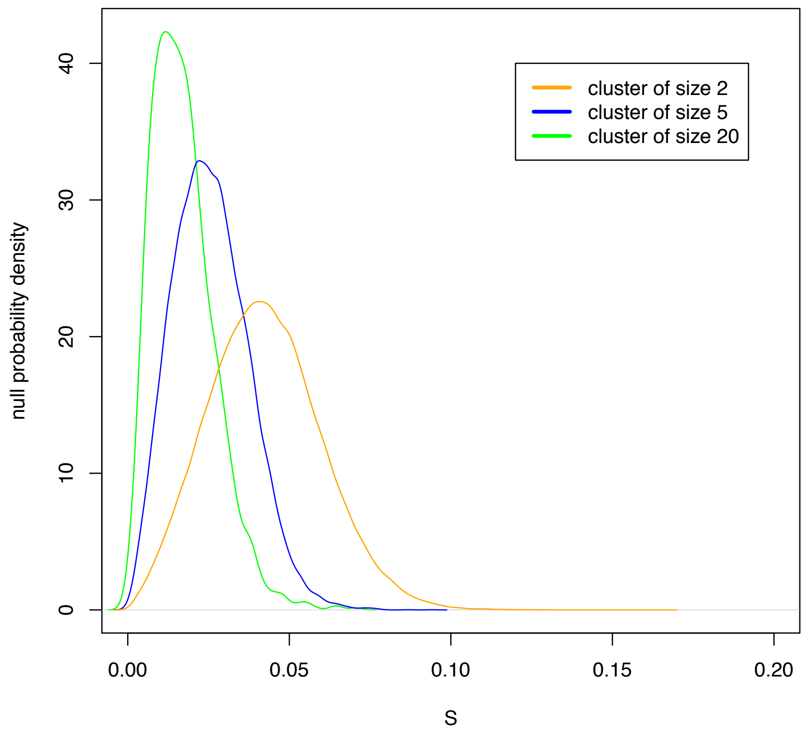

B: Simulated6

(1 - Pearson correlation) dissimilarity – average linkage combination

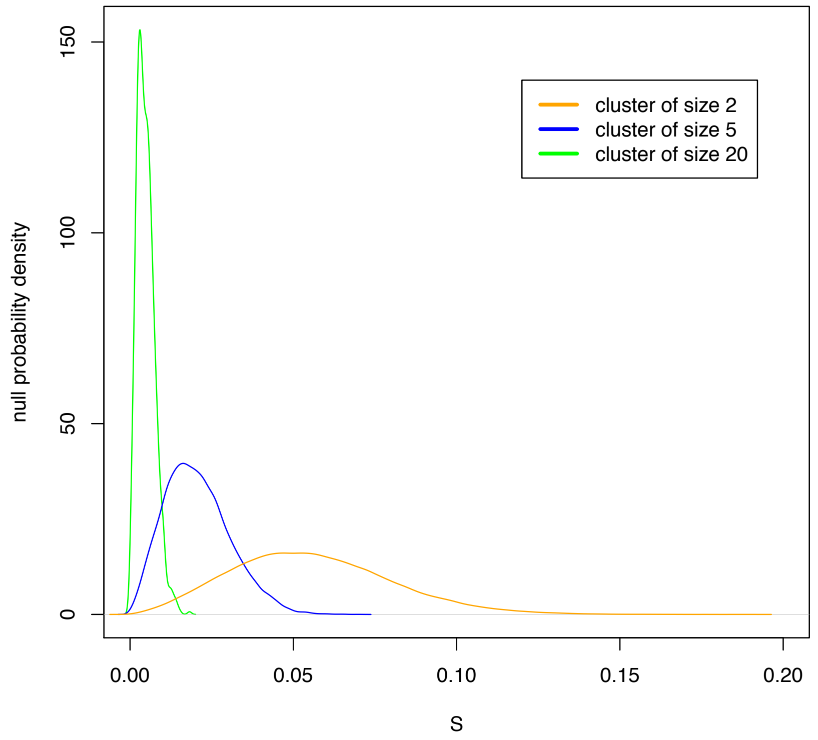

C: Leukemia
Euclidean dissimilarity – Ward linkage combination

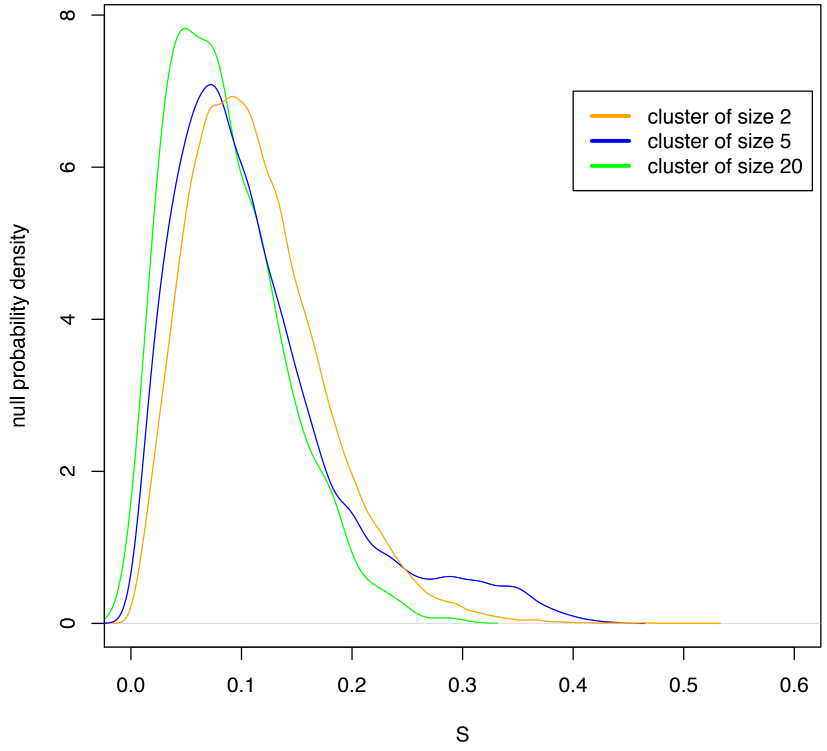

D: Leukemia
(1 - Pearson correlation) dissimilarity – average linkage combination

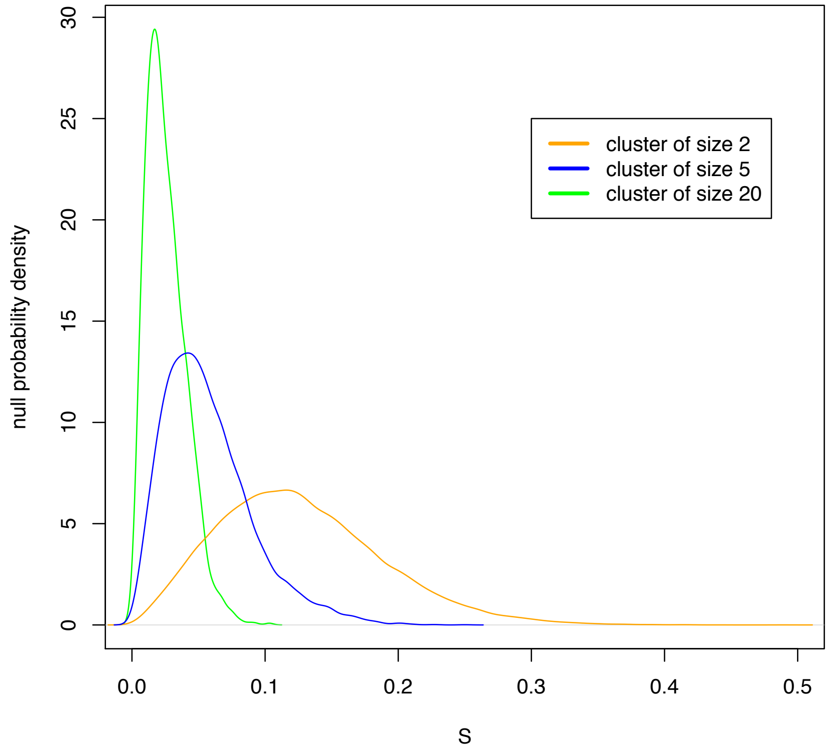

E: T10
Euclidean dissimilarity – Ward linkage combination

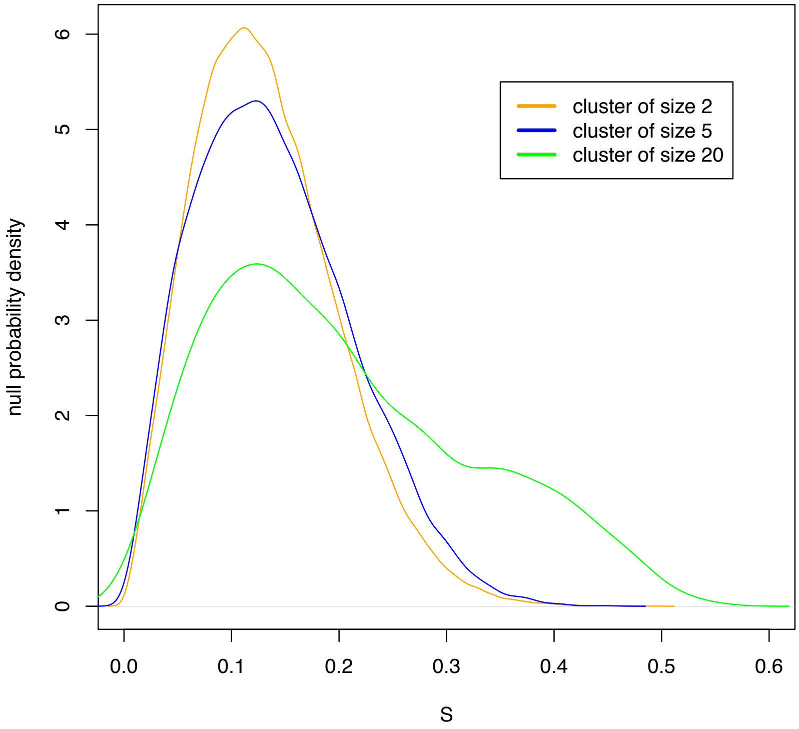

F: T10
(1 - Pearson correlation) dissimilarity – average linkage combination

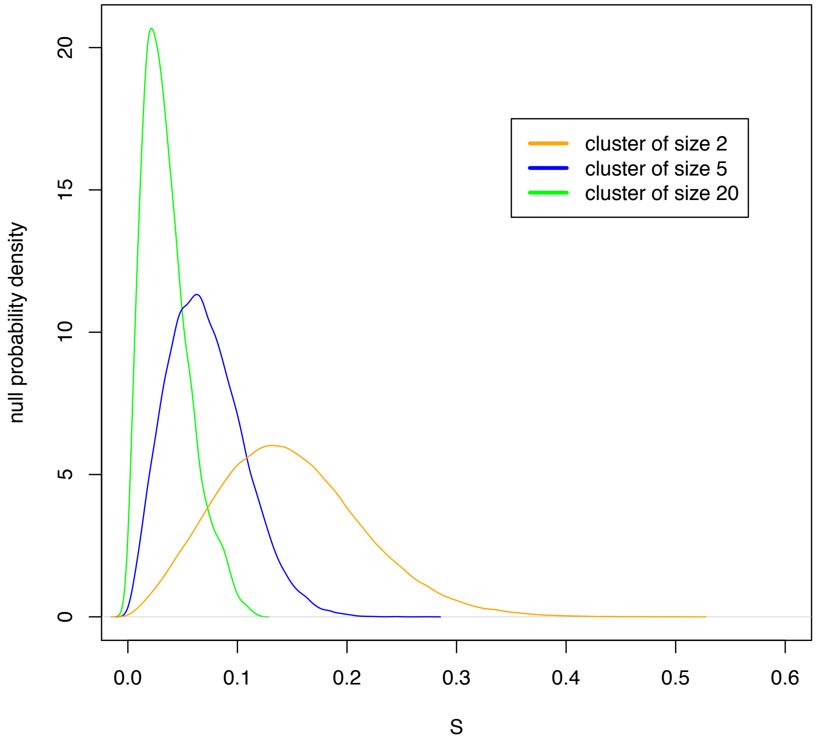

G: Organelles
(1 - Pearson correlation) dissimilarity – Ward linkage combination

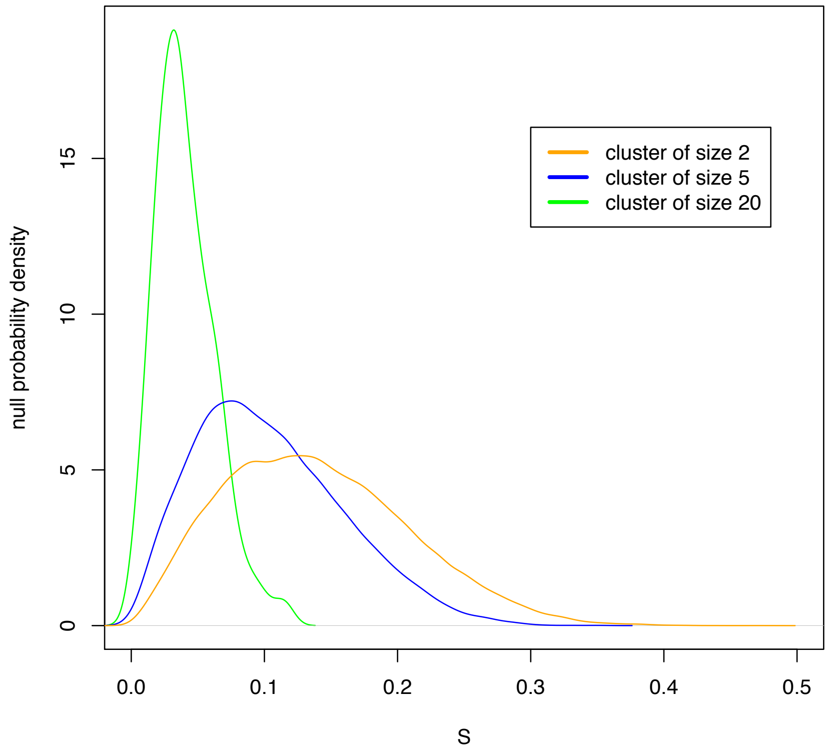

H: Organelles
(1 - Pearson correlation) dissimilarity – average linkage combination

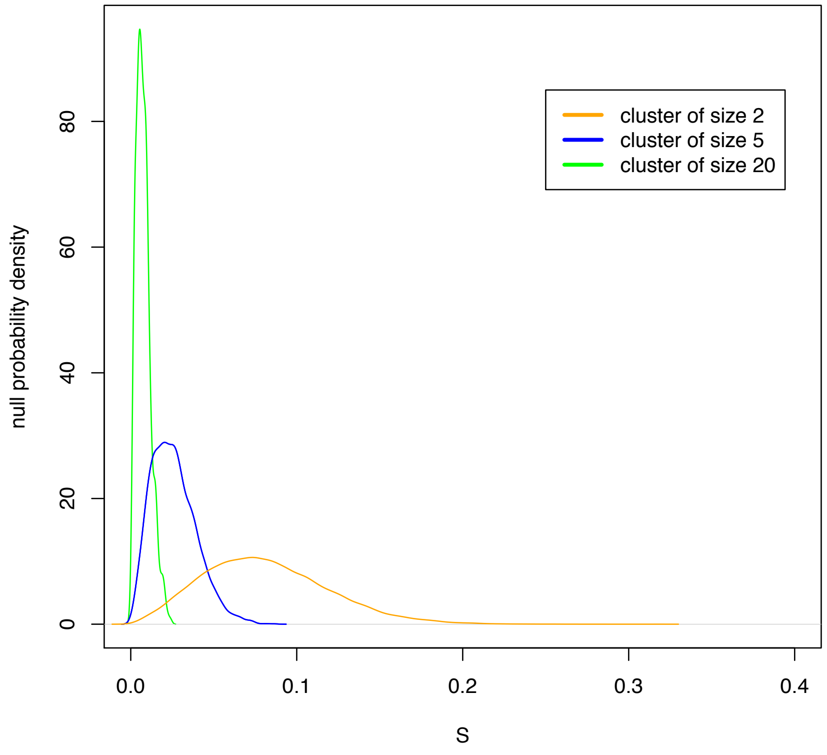

I: Chondrosarcoma

(1 - Spearman correlation) dissimilarity – Ward linkage combination

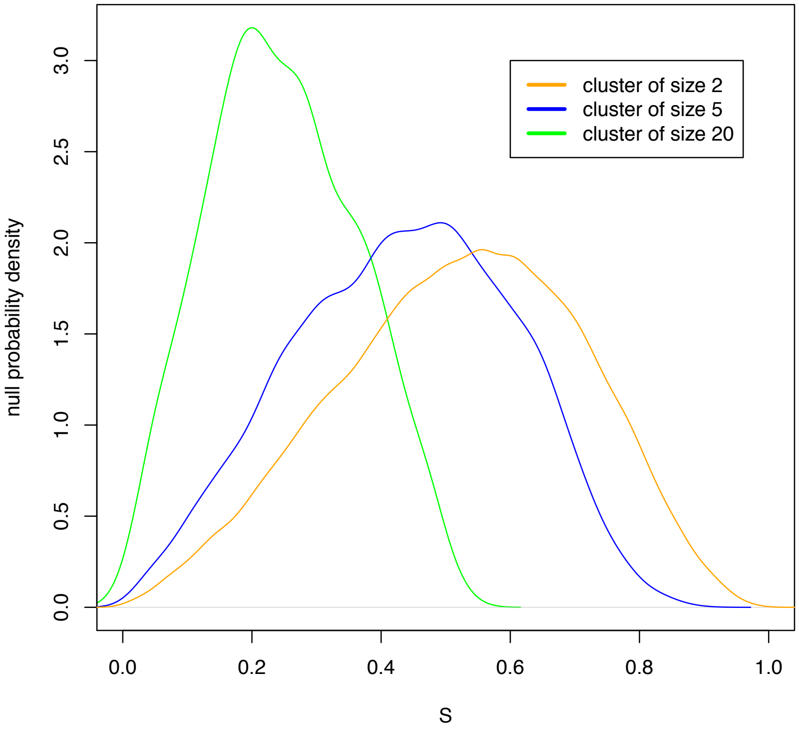

J: Chondrosarcoma

(1 - Kendall correlation) dissimilarity – average linkage combination

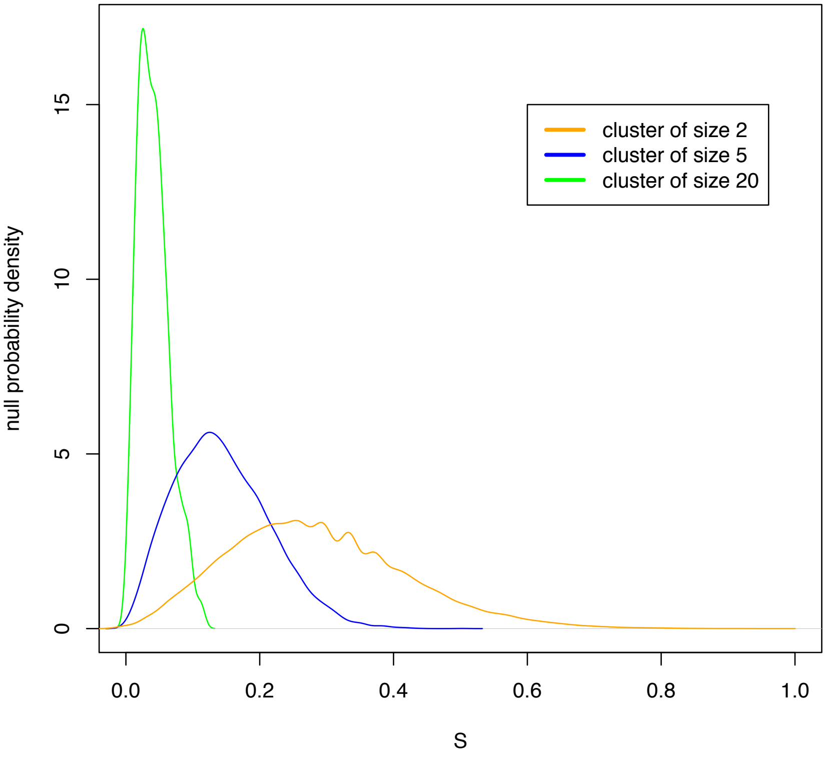

K: Chondrosarcoma

Manhattan dissimilarity – Ward linkage combination

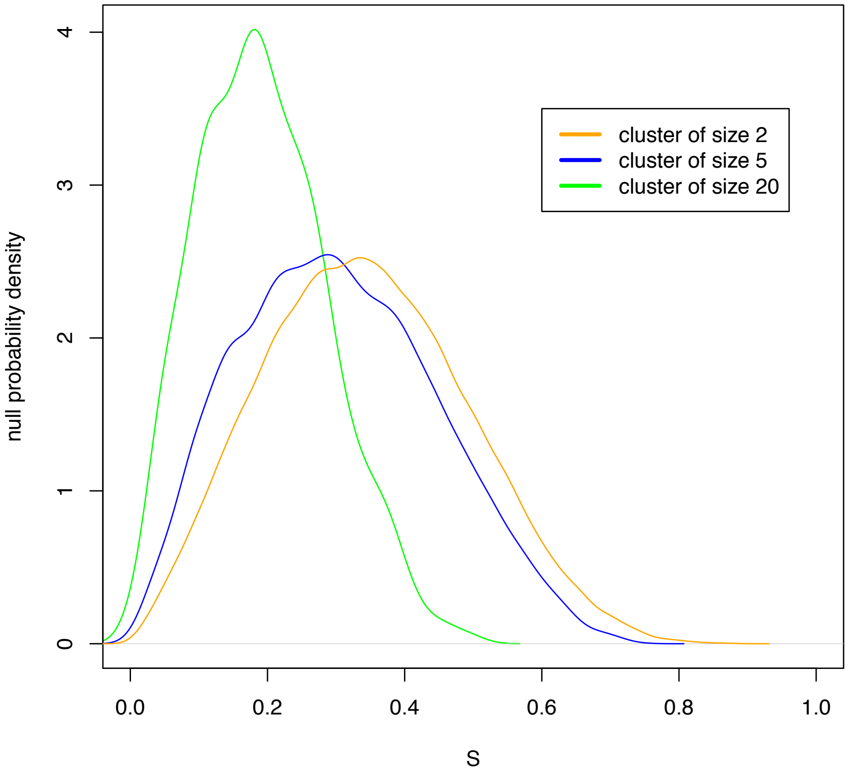

Figure S2

Empirical *p*-value estimates for tightness compared to EVT-based estimates.

Combined results for all tree nodes in all benchmark studies are shown. For each benchmark the combinations of dissimilarity and linkage are enumerated in the same order as they appear in Table 2. Displayed are the values corrected for hypothesis multiplicity (*cf* the Methods section). Empirical estimates are based on 1000×*N* randomizations each, *N* being the number of leaves. EVT estimates are based on 1000 randomizations each. If the empirical *p*-value estimate based on these 1000 randomization is large, the EVT algorithm defaults to this estimate. The corresponding points are shown by empty symbols of the appropriate shape and color. The diagonal dashed line indicates the identity. The vertical dashed line indicates the minimal multiplicity-corected empirical *p*-value $[1 - (1 - p_e)^{N-2}] / (n_r + 2)$, where *N* is the number of leaves and n_r is the number of randomizatons.

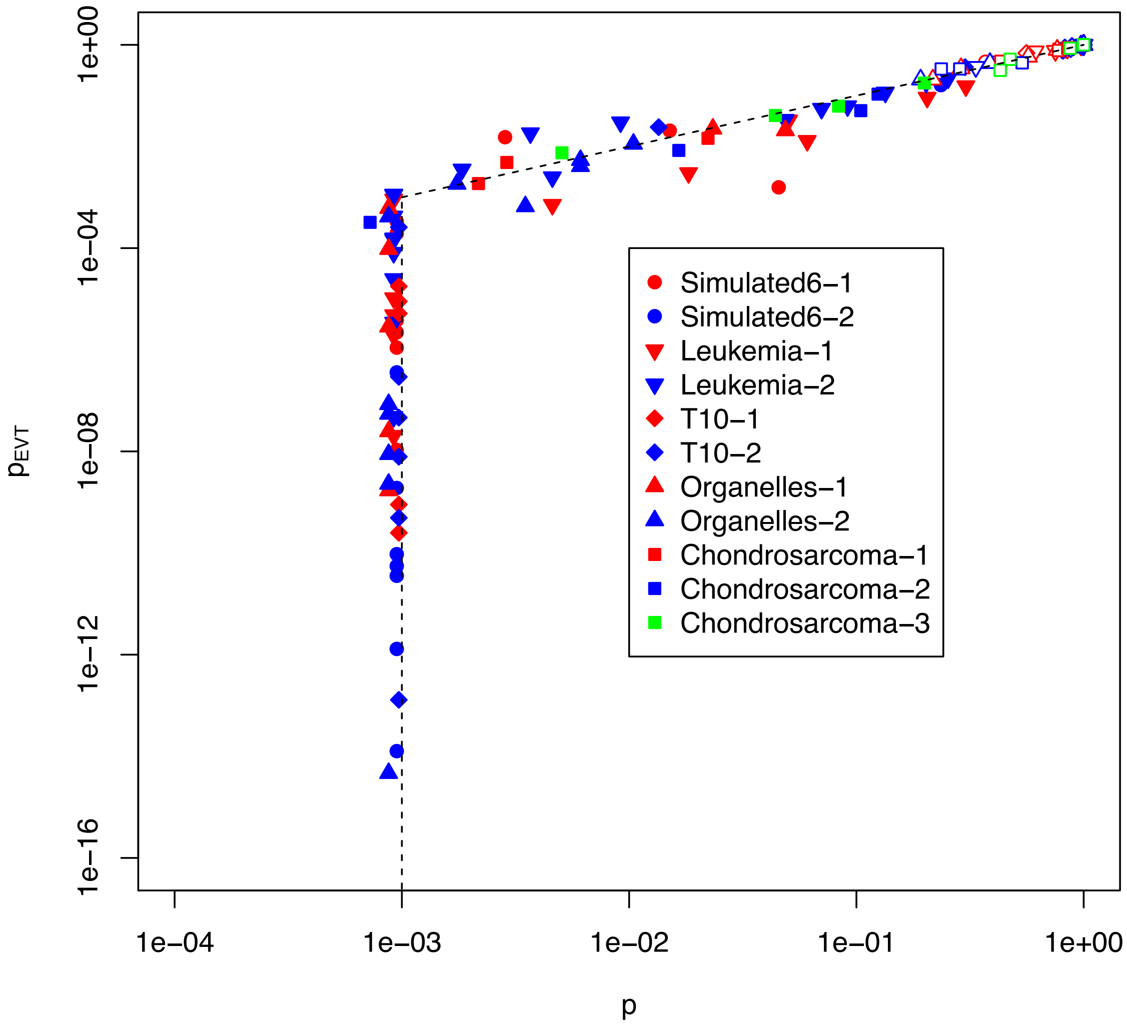

Table S1 Properties of the Simulated6 dataset*

genes	1-50	51-100	101-150	151-200	201-250	251-300	301-600
observations with up-regulated genes	1-8	9-20	21-30	31-45	46-50	51-60	
mean of up-regulated expressions	594	699	296	296	401	344	
regular mean	40	69	40	39	37	39	38
standard deviation	50	75	100	101	200	203	200

*Among the first 300 genes, each block of 50 genes comes from a normal distribution with parameters as tabulated, except for those observations within the class that these genes are up-regulated. Within each block the regular and the up-regulated distributions differ in the mean but have equal standard deviations. The values for the last 300 genes are drawn from the same normal distribution for all observations.
